# A family with Robertsonian translocation: a potential mechanism of speciation in humans

**DOI:** 10.1186/s13039-016-0255-7

**Published:** 2016-06-18

**Authors:** Jieping Song, Xi li, Lei Sun, Shuqin Xu, Nian Liu, Yanyi Yao, Zhi Liu, Weipeng Wang, Han Rong, Bo Wang

**Affiliations:** Genetics Laboratory, Hubei Maternal and Child Health Hospital, Wuhan, Hubei People’s Republic of China; Department of gastroenterology, Peking University Shenzhen Hospital, Shenzhen, Guangdong People’s Republic of China; Laboratory of Medical Genetics, Qinzhou Maternal and Child Health Care Hospital, Guangxi, People’s Republic of China; Shenzhen mental health center, Shenzhen Kangning Hospital, Shenzhen, Guangdong People’s Republic of China

**Keywords:** Chromosome karyotype, FISH, aCGH, Robertsonian translocation homozygosity, Evolution

## Abstract

**Background:**

Robertsonian translocations occur in approximately one in every 1000 newborns. Although most Robertsonian translocation carriers are healthy and have a normal lifespan, they are at increased risk of spontaneous abortions and risk of producing unbalanced gametes and, therefore unbalanced offspring. Here we reported a previously undescribed Robertsonian translocation.

**Case Presentation:**

We identified three Robertsonian translocation carriers in this family. Two were heterozygous translocation carriers of 45,XX or XY,der(14;15)(q10;q10) and their son was a homozygous translocation carrier of a 44,XY,der(14;15)(q10;q10), der(14;15)(q10;q10) karyotype. Chromosomal analysis of sperm showed 99.7 % of sperm from the homozygous translocation carrier were normal/balanced while only 79.9 % of sperm from the heterozygous translocation carrier were normal/balanced. There was a significantly higher frequency of aneuploidy for sex chromosome in the heterozygous translocation carrier.

**Conclusions:**

The reproductive fitness of Robertsonian translocation carriers is reduced. Robertsonian translocation homozygosity can be a potential speciation in humans with 44 chromosomes.

## Background

Robertsonian translocations, fusions between two acrocentric chromosomes, are the most common structural chromosomal rearrangements in humans and occur in approximately 1 in every 1000 newborns [1]. It occurs in the five acrocentric chromosomes, 13, 14, 15, 21, and 22, which have very small short arms that contain no unique genes. During a Robertsonian translocation, the participating chromosomes break at their centromeres and the long arms fuse to form a single chromosome with a single centromere. The short arms also join to form a smaller reciprocal product, which typically contains nonessential genes and is usually lost within a few cell divisions. This type of translocation is cytologically visible and can reduce chromosome number if the smaller chromosome that results from a translocation is lost over cellular divisions. However, the smaller chromosome lost may carry so few genes that it can be lost without an ill effect to the individual [2, 3].

When the translocation is balanced, the person with it is called a Robertsonian translocation carrier. As carriers are healthy and have a normal lifespanand, many never discover their unusual chromosome rearrangement. Therefore, the translocation can be passed down in families for many generations without anyone discovering. Robertsonian translocation carriers produce six types of gametes. At the end of meiosis I, segregation of the translocated and nontranslocated chromosomes from the two different chromosome pairs involved leads to the formation of either balanced gametes via alternate segregation or unbalanced gametes via adjacent segregation during anaphase [1, 2]. Meiotic tetravalent configuration tends to segregate in alternate way, resulting in preferential production of normal/balanced spermatozoa. However, certain percentages of unbalanced gametes derived from adjacent segregation are also produced, leading to the increased risk of miscarriage and chromosomally unbalanced fetus. In this study, we investigated a Robertsonian translocation family and analyzed the meiotic segregation patterns and interchromosomal effects in sperm from two Robertsonian translocation carriers.

## Case presentation

### Clinical information

**Fig. 1:**
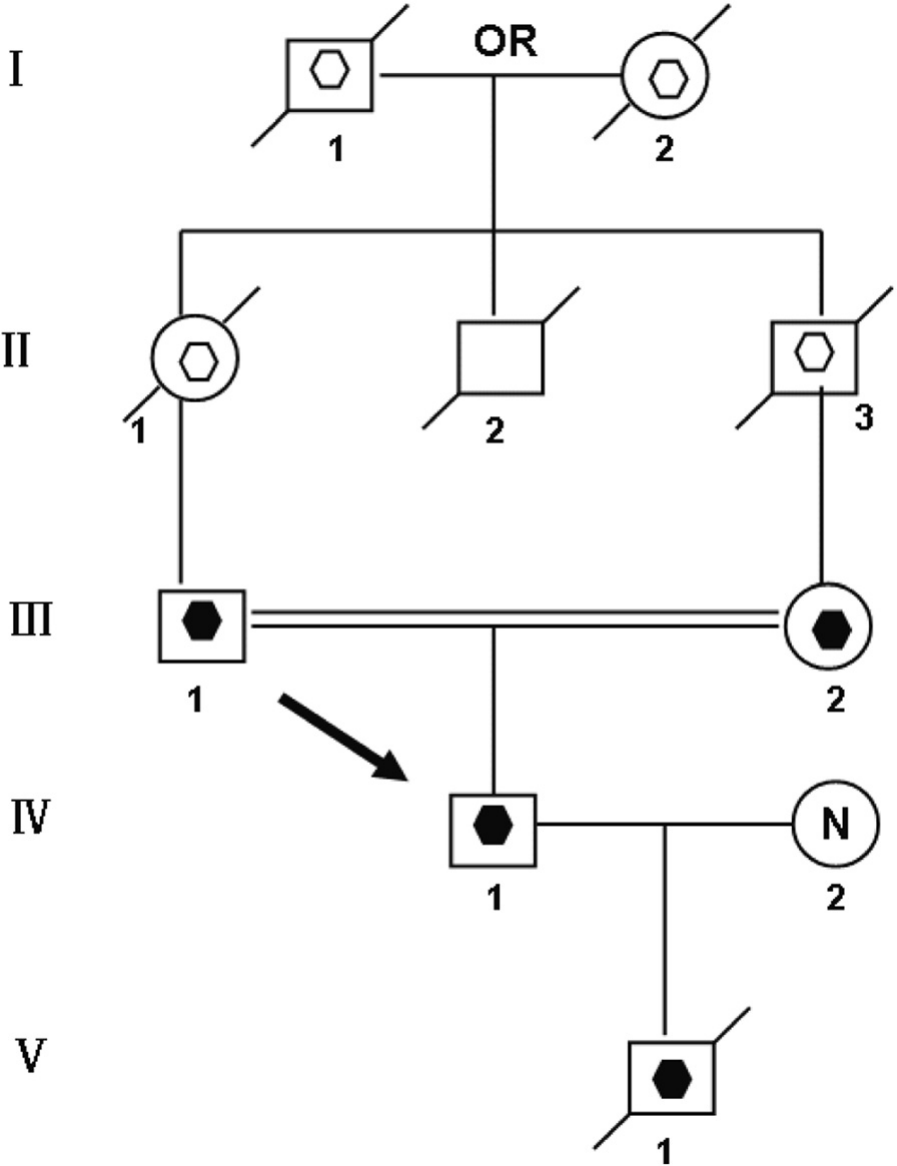
Pedigree of the family transmitting Rob translocation chromosome t(14; 15)(q10;q10) Open hexagon designates a presumed carrier of t(14; 15)(q10;q10). Filled hexagon designates a known carrier of t(14;15)(q10;q10). The proband, IV-1 (arrow), has disomy t(14;15)(q10;q10). The proband’s wife, IV-2, had a normal karyotype. Their deceased son, V-1, was a carrier of karyotype 45,XY,der(14;15)(q10;q10)

A 25-year-old Chinese man (IV-1) married to a non-consanguineous woman with normal chromosomes (IV-2). This couple had a son (V-1) who died at 6-month old without an autopsy. Previous chromosome analysis indicated V-1 had a 45,XY,der(14;15)(q10;q10) karyotype. The parents of the propositus (IV-1) are phenotypically normal first cousins (III-1, III -2). Their parents, a mutual uncle and both grandparents were not available for cytogenetic analysis (Fig. 1). Mental state examination was performed on IV-1 by a psychiatrist.

### Karyotype analysis

**Fig. 2:**
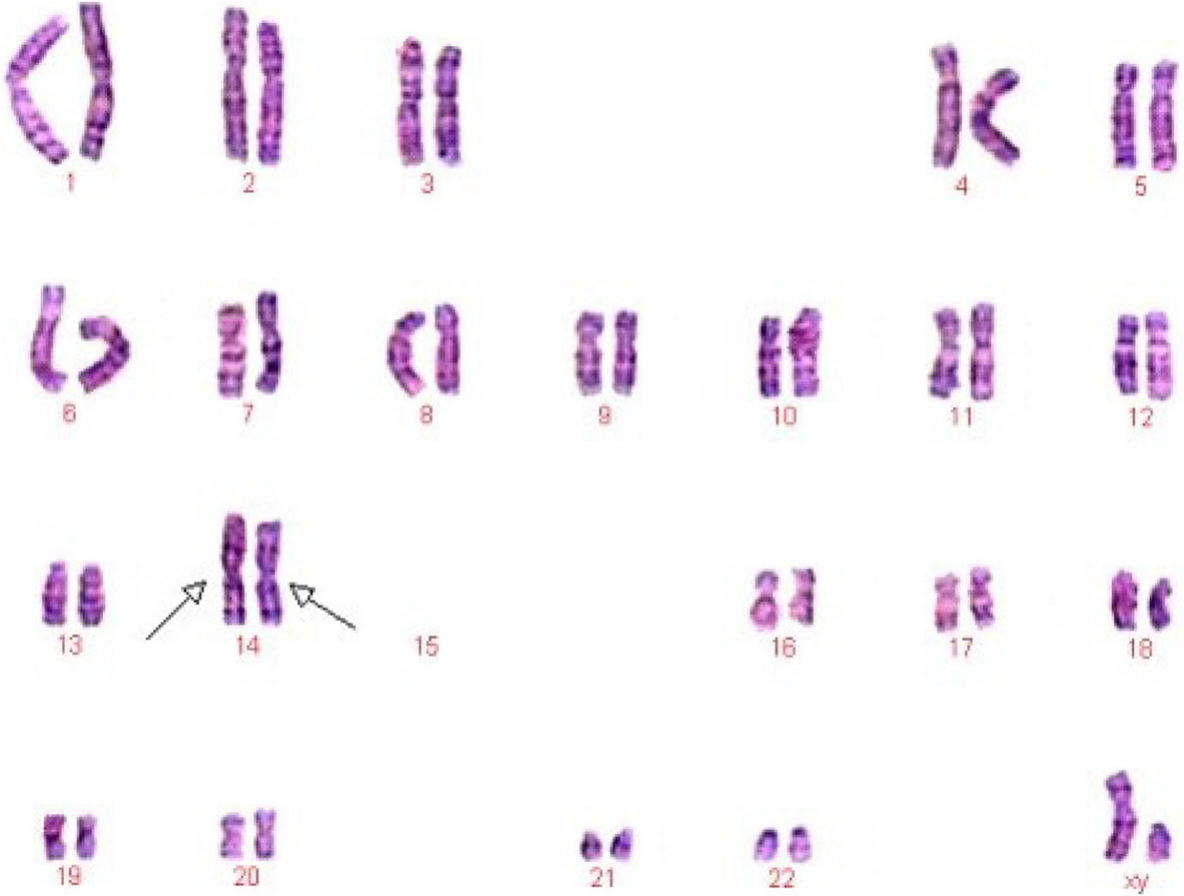
Karyotype of the proband with 44,XY,der(14;15)(q10;q10),der(14;15)(q10;q10) chromosomal constitution The arrows indicated disomy of chromosome der(14;15)

G-banding karyotype analysis was performed on all the four members in the family. The previously undescribed karyotype, 44,XY,der(14;15)(q10;q10), der(14;15)(q10;q10), was further authenticated by the Chinese Academic Committee of the State Key Laboratory of Medical Genetics (Fig. 2).

### Fluorescence in situ hybridization (FISH)

Sperm chromosomes from patient III-1 and IV-1 were analyzed by FISH. Semen samples were assessed for volume, concentration and motility according to the World Health Organization criteria. After semen analysis, progressively mobile sperm were isolated and washed twice in phosphate buffered saline (pH 7.4) and centrifuged at 1500 rpm for 11 min. Pellets were fixed with 5 ml of acetic acid/methanol mixture (1:3) for at least 30 min at 4 °C. Aliquots (40–50 μl) of the resulting suspension of nuclei were smeared on cold pre-cleaned slides. Nuclei decondensation was performed in 1 N NaOH for 2 min. The slides were then degraded by a graded ethanol series (70 %, 90 %, 100 %) and denatured with 0.25 % formamide in 2xSSC followed by overnight hybridization with a combination of commercially available probes. Two sets of probe mixtures were used in this study. Dual-color FISH was carried out using locusspecific probes (LSP) and Tel probes from Vysis (Vysis, Downers Grove, IL, USA). For patient IV-1 and III-1,TelVysion Probe 14q (D14S1420, Spectrum Red) was used for 14q32.33 and TelVysion Probe 15q(D15S120, Spectrum Green) was used for 15q26.3.

To investigate the presence of interchromosomal effect, triple-color FISH was performed using the second probe mixture, which consists of commercial satellite (DNA) probes from Vysis, including chromosomes 18, X and Y (CEP 18, Spectrum Blue/CEP X, Spectrum Green/CEP Y, Spectrum Red). Post-hybridization washes included 2 min in 0.4xSSC/0.3%NP-40 (pH = 7) at 72 °C, followed by 1 min in 2xSSC/0.1%NP-40 (pH = 7) at room temperature. Slides were covered with DAPI II (Vysis). Only intact spermatozoa bearing a similar degree of decondensation and clear hybridization signals were scored. All disrupted or overlapping spermatozoa were excluded from analysis. Slides with hybridization efficiency of 99 % and more were analyzed. 1,000 sperm nuclei were analyzed per patient.

### Array comparative genomic hybridization (aCGH)

Array comparative genomic hybridization (aCGH) has been introduced in clinical diagnosis to rapidly detect genomewide gains and losses with higher resolution [4]. It is a high throughput method which detects copy number changes to a resolution of even as low as 1 Kb. For aCGH analysis, DNA was extracted from peripheral blood [5]. aCGH analysis was performed on using the 8 × 60 K commercial arrays (Agilent Technologies, Santa Clara, CA, USA).

### Data analysis

The statistical analysis was performed using the SAS system (2002–2003, SAS Institute Inc., Cary, NC). Chi-squared test was used for meiotic segregation analysis. A probability value of less than 0.05 (*P* < 0.05) was considered to be statistically significant.

### Results

The karyotype analysis was performed on four members in the family and detected two different karyotypes of Robertsonian translocation. The results are shown in below. III-1 and III-2 were heterozygous while IV-1 was homozygous.

- III-1: 45,XY,der(14;15)(q10;q10)
- III-2: 45,XX,der(14;15)(q10;q10)
- IV-1: 44,XY,der(14;15)(q10;q10), der(14;15)(q10;q10)
- IV-2: 46,XX

Because the patient (IV-1) has a symptom of apathy, we referred him to see a psychiatrist for a thorough mental status examination. The patient had no psychiatric history and remained cooperative during the psychiatric interview. His thought processes were linear, logical, and consistently goal directed and his judgment was intact. Therefore, no psychiatric symptoms were observed. In addition, neurology consultation revealed no focal neurological deficits. The results of Craniocerebral Computed Tomography (CT) and lumbar puncture (LP) were normal. Electroencephalography (EEG) showed no signs of seizure activity.

**Fig. 3:**
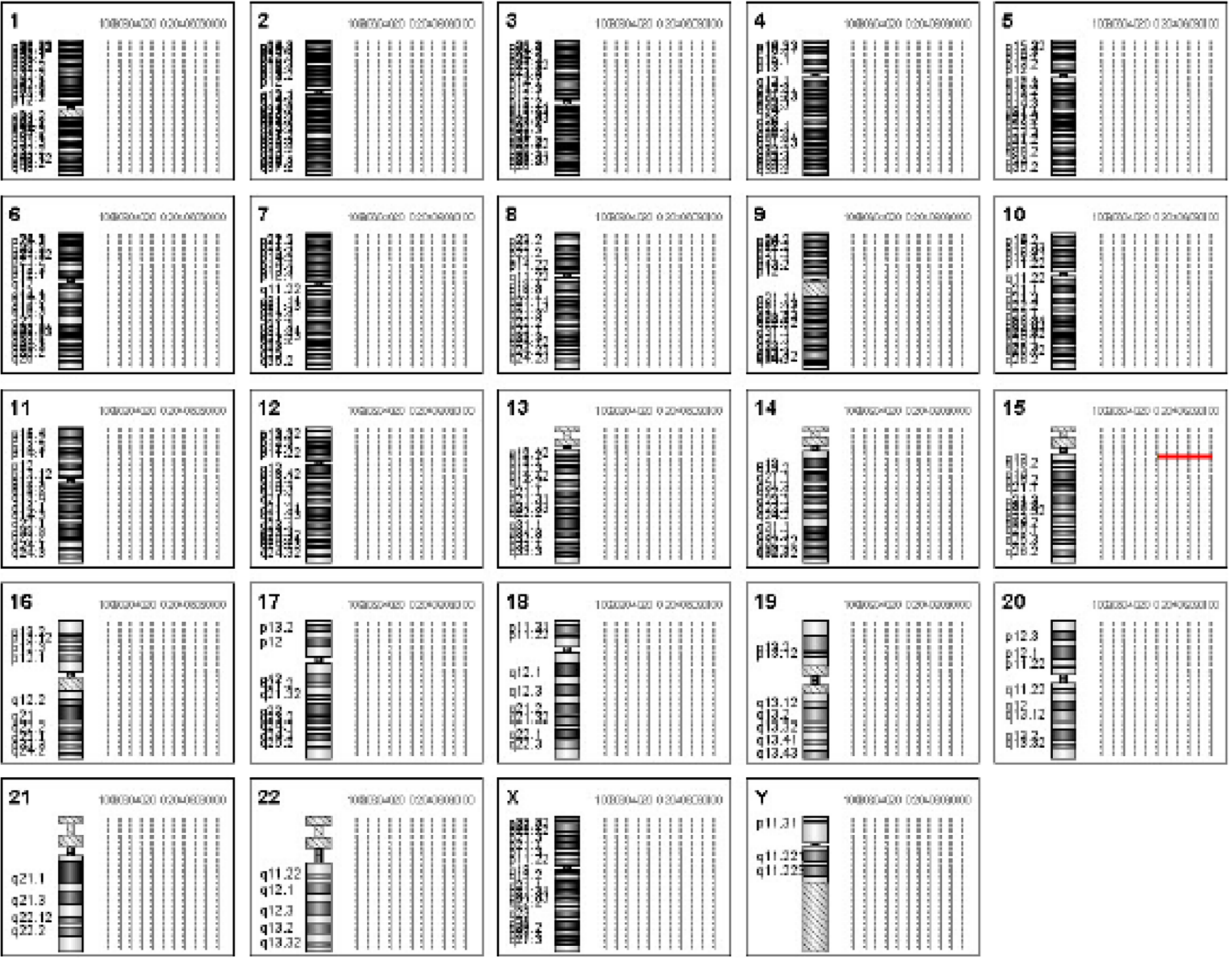
aCGH analysis of IV-1 revealed a 2.03 Mb duplication at chromosome 15 q11.1-q11.2 (arr 15q11.1q11.2(20,481,702-22,509,254)x4)

The aCGH analysis on IV-1 detected a 2.03 Mb duplication in the region of q11.1-q11.2 on chromosome 15 (Fig. 3). According to the Copy number variation database (CNVdb), this duplication is a chromosomal polymorphism in normal population. Because IV-1 is a is Homologous Robertsonian translocation carrier and his two derivative chromosomes came from the same Robertsonian translocation carrier, we speculated that each of the two derivative chromosomes should have a 2.03 Mb duplication in the region of q11.1-q11.2 on chromosome 15.

**Fig. 4:**
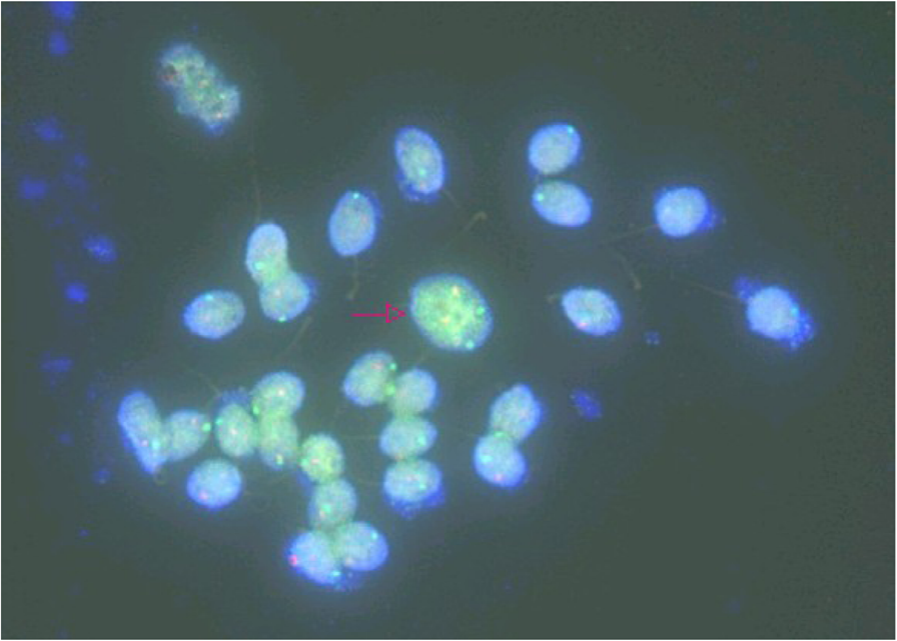
Sperm cells after hybridization with TelVysion 14q32.33 (Spectrum Red) and TelVysion 15q26.3 (Spectrum Green). The sperm with red arrow may be a diploid sperm

To analysis the sperm of III-1 and IV-1, semen samples from the two different Robertsonian translocation carriers (III-1 and IV-1) were assessed for volume, concentration and motility according to the World Health Organization criteria. The volume, concentration and mobility of sperm in III-1 are significantly reduced due to his old age. In contrast, they are all normal in IV-1 (Table 1). We further analyzed the meiotic segregation patterns in these two different Robertsonian translocation carriers. In the heterozygote Robertsonian translocation carrier (III-1), the frequency of normal/balanced spermatozoa resulting from alternate segregation was 79.9 % while the frequency of unbalanced spermatozoa resulting from adjacent segregation was 20.1 %, which is significantly higher than normal population [5–8] (Table 2). In contrast, in the homozygote Robertsonian translocation (IV-1), the frequency of balanced spermatozoa was 99.7 % while the frequency of unbalanced spermatozoa was only 0.3 %, which is similar to normal populations [5–8] (Table 2). These data demonstrated there nearly all sperm of Robertsonian translocation homozygotes were balanced while those of heterozygotes had a percentage of unbalanced. In addition, chromosome analysis of IV-1’s sperm showed either a 22,X,der (14;15) karyotype or 22,Y,der(14;15) karyotype supporting our speculation. As shown in Fig. 4, the arrow pointed a sperm with four fluorescence signals (two red and two green) suggesting this sperm has two derivative chromosomes of der(14;15). These data suggest a potential mechanism of speciation in humans via Robertsonian translocation.

**Table 1:** Cytogenetic and spermiologic results of III-1 and IV-1

Patient	Age (years)	Sperm concentration (×10^6^/ml)	Mobility (a + b) (%)
III-1	48	13	22
IV-1	25	56	53

**Table 2:** The number of spermatozoa scored, the alternate mode of segregation, incidence of sperm nullisomy, disomy and 3:0/diploid for the chromosomes involved in the Rob translocation in two Rob translocation carriers

Segregation modes	III-1	IV-1
normal or balanced	799	997
nullisomy 14	41	2
disomy 14	49	0
nullisomy 15	55	0
disomy 15	45	0
3:0 or diploid	11	1
Total	1000	1000

The influence of translocated chromosomes on the synapses and disjunction of other chromosomes is called an interchromosomal effect (ICE) [9]. To determine whether ICE was present, the nullisomy, disomy and diploid rates for chromosomes 18, X and Y were analyzed in both Robertsonian translocation carriers (Table 3). In the heterozygote Robertsonian translocation carrier (III-1), there was a significant increase in the rate of sex chromosome aneuploidy and diploid. In contrast, in the homozygote Robertsonian translocation (IV-1), the incidence of spermatozoa with nullisomy, disomy and diploid for the sex chromosomes remained similar to normal populations (Table 3) [5–8]. This data suggest that ICE only presents in certain Robertsonian translocation carriers.

**Table 3:** Incidence of sperm nullisomy, disomy and diploid for chromosomes 18, X and Y in two Rob translocation carriers

Segregation modes	III-1	IV-1
normal or balanced	949	994
nullisomy 18	3	0
disomy 18	2	1
nullisomy Sex chromosome	15	2
disomy Sex chromosome	10	3
3:0 or diploid	21	0
Total	1000	1000

### Discussion

Rob translocation carries are phenotypically normal since they involve loss only of short arm material. Many such rearrangments in natural populations of different species are known. Robertsonian rearrangments can increase polymorphisms in a species, provide material for natural selection and even lead to speciation [10, 11]. It has been argued that the types of chromosome rearrangments that occur as polymorphisms or as fixed permanent heterozygotes invariably involve meta- or submetacentric chromosomes. Those that distinguish species and serve to isolate these species involve telocentric or acrocentric chromosomes, which are self-sterilizing [12]. In our study, IV-1, who is a homologous Robertsonian translocation carrier, was healthy and had a balanced chromosomal complement. Assessment of a semen sample from showed normal sperm morphology and motility. More importantly, 99.7 % of his sperm are normal or balanced (Table 2) and there was no interchromosomal effect (Table 3).

Multicolor FISH on decondensed sperm nuclei allows for a rapid analysis of meiotic segregation in sperm of translocation carriers, providing information on the exact amount of normal/balanced sperm [13]. Such information is undoubtedly important not only for basic cytogenetic research but also for reproductive counseling of Robertsonian translocation carriers. t(14;15) are rare Robertsonian translocations. Our data confirmed previous studies showing that alternate segregation was more frequently than adjacent segregations in Robertsonian translocations carriers [5, 8]. However, the meiotic segregation patterns and interchromosomal effects in two Robertsonian translocation carriers are different (Table 2). These findings are consistent with previously published data [14–16]. The high prevalence of the alternate segregation is presumably due to cis-configuration of the trivalent during meiosis, which favors an alternate segregation in all Robertsonian translocations [2]. Although a Robertsonian translocation carrier has a full genetic complement, their productive fitness is reduced due to high probability of genetically imbalanced gametes.

The interchromosomal effect could be explained by the formation of heterosynapses between chromosomes involved in the translocation and the sex vesicle, which could also involve other chromosomes [17]. ICE remains controversial in the literature. Some publications have indicated its presence with Robertsonian translocation [18, 19] while others demonstrated no evidence of this phenomenon [4, 20]. In our study, we observed higher incidence of aneuploidy for sex chromosomes in spermatozoa in the heterozygote Robertsonian translocation but not the homozygote Robertsonian translocation suggesting that ICE only presents in some Robertsonian translocation carriers.

Robertsonian rearrangements are common chromosomal changes that can lead to rapid and efficient reproductive isolation between karyotypically similar populations, especially when many Robertsonian metacentric chromosomes display monobrachial homologies [10]. Homozygous Robertsonian translocations have reported by several groups [21–23] and systemically reviewed by the O’Neill group [24]. Dallapiccola et al. [21] reported a fetus with two t(14;21) chromosomes. The related parents were heterozygous for the same translocation. Martinez et al. [22] reported three adult siblings homozygous for t(13;14). Their parents were first-cousins and both were heterozygous carriers. Rajangam et al. [23] found a unique DS karyotype 45, XY, der(14;21)pat, der(14;21)mat, +21mat. Although translocation heterozygosity is associated with meiotic disturbances, which will lead to infertility and subfecundity, it should not have any effect on meiosis. Here we reported a man with a 44,XY,der(14;15)(q10;q10),der(14;15)(q10;q10) karyotype. Robertsonian translocation may provide material for evolution. Long term isolation of a group of individuals who are homozygous for a particular Robertsonian translocation chromosome could theoretically lead to the establishment of a new human subspecies having a full genetic complement in 44 chromosomes. Although karyotypic differences between species have long been recognized, the question of whether these mutations play a causal role in speciation remains unanswered [25–27]. In our study, all sperm of Robertsonian translocation homozygotes were balanced while those of heterozygotes had a percentage of unbalanced ones implicating of a potential of the homozygosity of Robertsonian translocations for speciation.

## Conclusions 

To conclude, in this study we reported a previously described Robertsonian translocation karyotype. The homozygosity of Robertsonian translocation for speciation may be a potential mechanism of speciation in Humans.

## Abbreviations

aCGH, Array comparative genomic hybridization; CT, Craniocerebral Computed Tomography; EEG, Electroencephalography; FISH, Fluorescence in situ hybridization;; ICE, interchromosomal effect; LP, lumbar puncture
